# Obstructive sleep apnoea but not central sleep apnoea is associated with left ventricular remodelling after acute myocardial infarction

**DOI:** 10.1007/s00392-020-01684-z

**Published:** 2020-06-09

**Authors:** Christoph Fisser, Kristina Götz, Andrea Hetzenecker, Kurt Debl, Florian Zeman, Okka W. Hamer, Florian Poschenrieder, Claudia Fellner, Stefan Stadler, Lars S. Maier, Michael Pfeifer, Stefan Buchner, Michael Arzt

**Affiliations:** 1 Department of Internal Medicine II, University Medical Centre Regensburg, Franz-Josef-Strauß-Allee 11, 93053 Regensburg, Germany; 2 Department of Pneumology, Donaustauf Hospital, Donaustauf, Germany; 3 Centre for Clinical Studies, University Medical Centre Regensburg, Regensburg, Germany; 4 Department of Radiology, University Medical Centre Regensburg, Regensburg, Germany; 5 Department of Internal Medicine, Cham Hospital, Cham, Germany

**Keywords:** Myocardial infarction, Sleep apnoea, Sphericity, Cardiac remodelling, Wall thickness, Aneurysm, Cardiac magnetic resonance

## Abstract

**Obejctive:**

Obstructive sleep apnoea (OSA) increases left ventricular transmural pressure more than central sleep apnoea (CSA) owing to negative intrathoracic pressure swings. We tested the hypothesis that the severity of OSA, and not CSA, is therefore associated with spheric cardiac remodelling after acute myocardial infarction.

**Methods:**

This sub-analysis of a prospective observational study included 24 patients with acute myocardial infarction who underwent primary percutaneous coronary intervention. Spheric remodelling, calculated according to the sphericity index, was assessed by cardiac magnetic resonance imaging at baseline and 12 weeks after acute myocardial infarction. OSA and CSA [apnoea-hypopnoea index (AHI) ≥ 5/hour] were diagnosed by polysomnography.

**Results:**

Within 12 weeks after acute myocardial infarction, patients with OSA exhibited a significant increase in systolic sphericity index compared to patients without sleep-disordered breathing (no SDB) and patients with CSA (OSA vs. CSA vs. no SDB: 0.05 ± 0.04 vs. 0.01 ± 0.04 vs. − 0.03 ± 0.03, *p* = 0.002). In contrast to CSA, the severity of OSA was associated with an increase in systolic sphericity index after accounting for TIMI-flow before percutaneous coronary intervention, infarct size, pain-to-balloon-time and systolic blood pressure [OSA: B (95% CI) 0.443 (0.021; 0.816), *p* = 0.040; CSA: 0.193 (− 0.134; 0.300), *p* = 0.385].

**Conclusion:**

In contrast to CSA and no SDB, OSA is associated with spheric cardiac remodelling within the first 12 weeks after acute myocardial infarction. Data suggest that OSA-related negative intrathoracic pressure swings may contribute to this remodelling after acute myocardial infaction.

**Graphic abstract:**

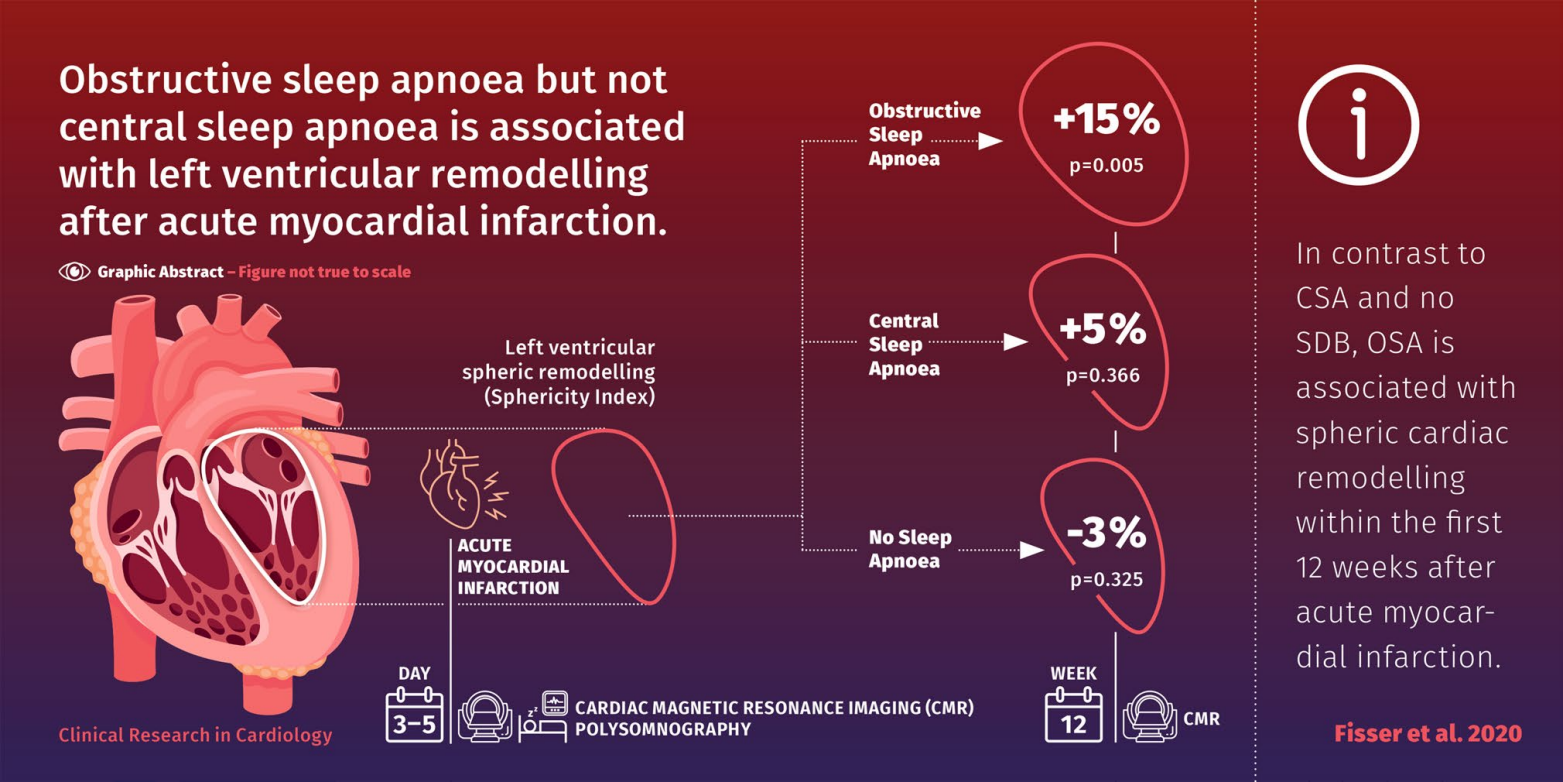

**Electronic supplementary material:**

The online version of this article (10.1007/s00392-020-01684-z) contains supplementary material, which is available to authorized users.

## Introduction

Over recent decades, patient survival after ST-elevation myocardial infarction (STEMI) has improved tremendously due to innovative therapeutic advances in the treatment and management of this condition [1]. However, postinfarction heart failure resulting from left ventricular remodelling processes remains a major predictor of prognosis [2].

Most cardiac aneurysms after STEMI affect the anterior wall of the left ventricle and are a strong predictor for heart failure and surval after myocardial infarction [3]. Infarct expansion—thinning and dilatation of the left ventricle after myocardial infarction—transforms the helical structure of the apex into a more spherical shape [4–6]. Spheric cardiac remodelling can be measured by the sphericity index [7] and is characterised by a decline in left ventricular ejection fraction, and a decrease in 10-year survival after myocardial infarction [5, 8].

Sleep-disordered breathing (SDB) manifests in two distinct types, obstructive and central sleep apnoea (OSA and CSA, respectively) [9]. The hallmark of OSA is breathing efforts against the occluded pharynx, leading to negative inspiratory pressure swings [10] and to a significantly increased left ventricular transmural wall pressure. Tkacova et al. demonstrated in a sample of patients with chronic heart failure and reduced ventricular ejection fraction that treatment of severe OSA reduces left ventricular transmural wall pressure [11].

CSA is characterised by periods of hyperventilation, followed by a reduction in pCO_2_ below the apnoea threshold and leads to intermittent hypoxaemia without inspiratory efforts and negative intrathoracic pressure. Therefore, in contrast to OSA, CSA may result in a smaller effect on afterload [12] and a slightly increased stroke volume [13].

The effects of OSA on left ventricular wall pressure, especially in patients with heart failure [14], may promote spheric cardiac remodelling during the vulnerable early phase after myocardial infarction to a greater extent than the effects of CSA. However, an association between SDB, classified into CSA and OSA, and spheric cardiac remodelling has not yet been reported. Therefore, we tested the hypothesis that in patients with acute myocardial infarction, the severity of OSA, but not CSA, is independently associated with spheric remodelling, assessed by cardiac magnetic resonance (CMR).

## Methods

A sub-analysis of a prospective observational study [15–18] in patients with acute myocardial infarction was conducted at the University Medical Centre Regensburg (Regensburg, Germany). Details of the study design have been published previously [15–18].

### Patients

The inclusion criteria for this sub-analysis were as follows: patients aged 18–80 years with acute myocardial infarction and percutaneous coronary intervention, who were treated at the University Medical Centre Regensburg (Regensburg, Germany) within 24 h of symptom onset. Key exclusion criteria included previous myocardial infarction or previous myocardial revascularisation, indication for surgical myocardial revascularisation, cardiogenic shock, implantation of a cardiac device, or other contraindications for CMR, known treated SDB, and impractical patient follow-up (e.g., distant place of residence, language barrier).

Between March 2009 and March 2012, 252 consecutive patients who underwent percutaneous coronary intervention to treat acute myocardial infarction were screened. 74 patients were eligible for the prospective observational study [16]. 50 patients were excluded from this sub-analysis owing to missing CMR (*n* = 7), missing polysomnography (*n* = 1), or myocardial infarction other than left anterior descending (*n* = 42). The final sub-analysis included 24 patients, who could be divided into three cohorts (no SDB, OSA and CSA).

### Study design

The study protocol was reviewed and approved by the local institutional ethics committee (Regensburg, 08-151) and the research was conducted according to the Declaration of Helsinki and Good Clinical Practice. All patients signed written informed consent prior to enrolment.

Eligible patients underwent an overnight in-laboratory sleep study (polysomnography − PSG) 3–5 days after percutaneous coronary intervention [15–18].

Clinical management and medication were at the discretion of the responsible physician according to current best practice. Patients were divided into two groups based on the following specifications: without SDB (AHI < 5 events per hour) and with SDB (AHI ≥ 5 events per hour). Patients with SDB were additionally separated into a CSA cohort (more central than obstructive episodes) and an OSA cohort (more obstructive than central episodes). None of the patients received positive airway pressure therapy within the first 12 weeks after acute myocardial infarction.

### Polysomnography (PSG)

All patients underwent PSG using standard polysomnographic techniques (Alice System; Respironics, Pittsburgh, PA, USA) [15–18]. The sleep laboratory is located on the cardiology ward at the University Medical Centre Regensburg, to which participants with acute myocardial infarction were admitted. The median time to PSG after acute myocardial infarction was 3 days. Respiratory efforts were measured by means of respiratory inductance plethysmography, and airflow was measured with a nasal pressure cannula. Sleep stages, arousals, apnoeas, and hypopnoeas were determined according to the criteria of the American Academy of Sleep Medicine [9] by an experienced sleep technician blinded to the clinical data. Apnoea was defined as cessation of inspiratory airflow for ≥ 10 s. Hypopnoea definition A was used (≥ 30% reduction in airflow and ≥ 4% desaturation) [9]. In addition, hypopnoeas were classified as obstructive if there was out-of-phase motion of the ribcage and abdomen, or if airflow limitation was present. In order to accurately distinguish between obstructive and central hypopnoeas without using an oesophageal balloon, we applied additional criteria, such as flattening, snoring, paradoxical effort movements, arousal position relative to hypopnoeas and associated sleep stage (rapid eye movement (REM)/non-REM) [19]. Apnoea-hypopnoea index (AHI) was defined as the number of central (cAHI) or obstructive (oAHI) apnoea and hypopnoea episodes per hour of sleep [20]. CSA was defined as cAHI/AHI > 50% and OSA as cAHI/AHI ≤ 50%.

### CMR acquisition protocol

Details of the CMR acquisition protocol have been published previously [15–18]. CMR was performed using a clinical 1.5-T scanner (Avanto; Siemens Healthcare Sector, Erlangen, Germany) with a 32-channel phased-array receiver coil. Cine images in standard short axis planes, two chamber and four chamber views (slice thickness 8 mm, inter-slice gap 2 mm, repetition time 60.06 s, echo time 1.16 s, flip angle 60 °, field of view 300 × 300 mm, matrix size 134 × 192 pixels, readout pixel bandwidth 930 Hz per pixel) was performed using acquisition of steady-state free precession. Delayed enhancement images were obtained using a segmented inversion recovery steady-state free precession technique (slice thickness 8.0 mm, inter-slice gap 2 mm, repetition time 1 RR interval, echo time 1.48 ms, flip angle 60 °, field of view 360 × 360 mm, matrix size 128 × 256) and acquired 10–15 min after injection of gadolinium.

### CMR image analysis

Details of CMR image analysis have been published previously [15–18]. Wall thickness and cardiac volumes were evaluated at baseline and 12 weeks after the myocardial infarction. Planimetric studies were conducted in the serial short axis slices. The basal and apical layers of myocardial muscle were determined visually. The epi- and endocardial textures in all other layers were marked semi-automatically. Wall thickness and thickening was automatically assessed by the AHA 17 segment model [21]. By definition, eight segments relate to the left anterior descending artery (basal anterior, basal anterospetal, mid-anterior, mid-anteroseptal, apical anterior, apical septal, apical lateral and apical). Variation owing to different coronary artery supply is possible. To evaluate left ventricular size, the distance between the apex and the mitral valve area in 2- and 4-chamber-view was measured manually in systole and diastole.

**Fig. 1:**
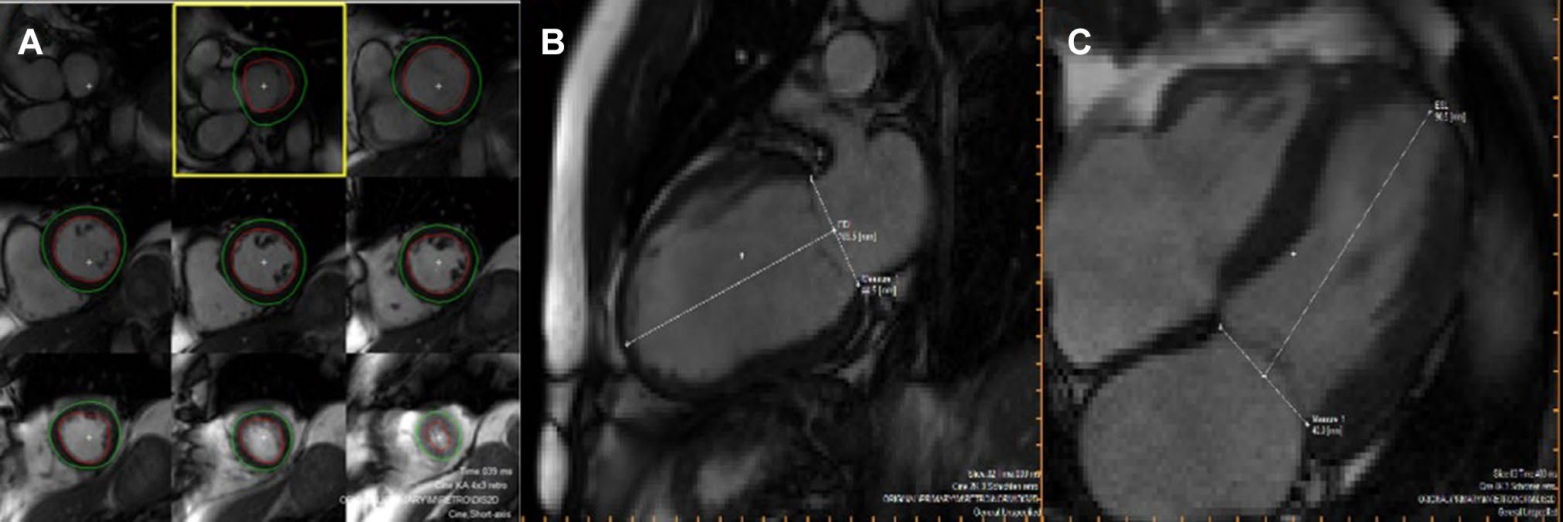
Example of magnetic resonance imaging of the left ventricle: **a** Planimetry, **b**, **c** size of left ventricle

To assess wall thickness the sum of the related segments was computed and the arithmetic mean was calculated (Fig. 1). The difference between baseline and follow-up readings was calculated. Quality control of measurements was overseen by two experienced cardiologists.

### Sphericity index

The sphericity index is an approved index used to describe the degree of left ventricular geometric abnormality in anterior wall myocardial infarction [7, 22]. It quantifies the extent of cardiac remodelling after myocardial infarction according to the following formula [22]:

$$\mathrm{S}\mathrm{p}\mathrm{h}\mathrm{e}\mathrm{r}\mathrm{i}\mathrm{c}\mathrm{i}\mathrm{t}\mathrm{y} \mathrm{I}\mathrm{n}\mathrm{d}\mathrm{e}\mathrm{x}= \frac{\mathrm{L}\mathrm{V} \mathrm{v}\mathrm{o}\mathrm{l}\mathrm{u}\mathrm{m}\mathrm{e}}{\frac{4}{3} * \pi * {\left(\frac{\mathrm{L}\mathrm{V} \mathrm{l}\mathrm{e}\mathrm{n}\mathrm{g}\mathrm{t}\mathrm{h}}{2}\right)}^{3}}$$

An upper limit of 0.29 in diastole is considered non-pathologic [7]. Calculation was performed at baseline and at 12-weeks follow-up.

### Recordings of 24-h blood pressure

Blood pressure measurement was conducted with the noninvasive portable SpaceLabsTM90207 (OSI Systems Inc) device. Patients were advised to keep calm during the measurements. Average values for blood pressure were analyzed for each hour and for the 24-h period.

### Statistical analysis

Unless otherwise indicated, descriptive data are expressed as means ± SD or as frequencies and percentages of each category. The groups of patients with no SDB, CSA and OSA were compared using one-way analysis of variance (ANOVA) for continuous variables, the Chi-square test for categorical variables and the Kruskal–Wallis Test for ordinal and skewed variables. Bonferroni test was used for post-hoc analysis. Linear regression models were calculated to assess the predictive value of CSA and OSA with respect to sphericity index. One multiple linear regression model was adjusted for infarct size, TIMI flow pre-percutaneous coronary intervention and pain-to-balloon-time, and one model was adjusted for the demographic variables age, sex and BMI. Scatter plots with regression lines were used to visualise the relationship between variables. All reported *p* values are two-sided, with 0.05 considered the threshold for statistical significance. Data entry and data analysis were performed with the software package SPSS 25.0 (Chicago, EUA).

## Results

### Patient characteristics

**Fig. 2:**
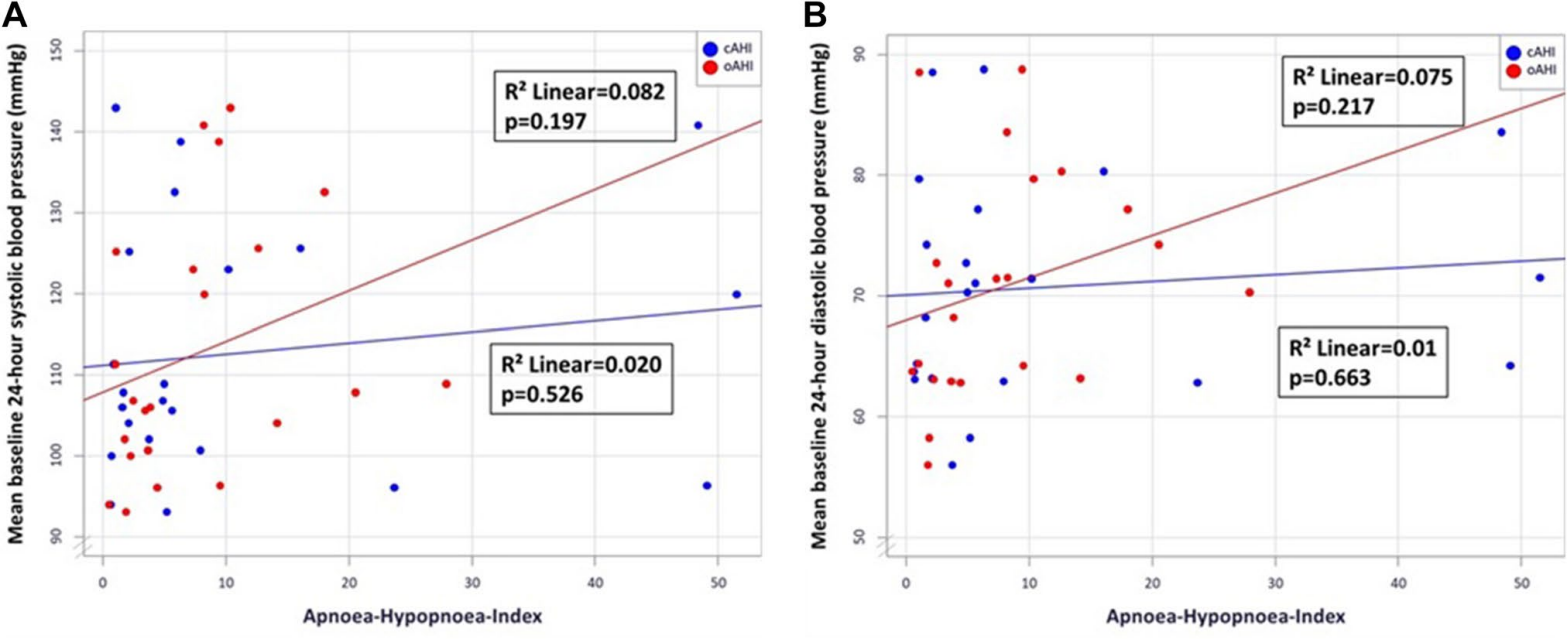
**a** Scatter plot—mean baseline 24-h systolic blood pressure and obstructive/central apnoea-hypopnoea index. **b** Scatter plot—mean baseline 24-h diastolic blood pressure and obstructive/central apnoea-hypopnoea index

The characteristics of the patients at baseline were well balanced among groups (Table 1). The median age among all the patients was 49 ± 10, 57 ± 8 and 54 ± 8 years (no SDB, CSA, OSA, respectively), and 88% of the patients were male. The prevalence of hypertension, diabetes, smoking status, hypercholesterinaemia was similar in the trial groups. All patients received standard pharmacological therapy after acute myocardial infarction. Mean 24-h blood pressure in systole and diastole did not differ significantly between the CSA and the OSA groups (Table S1). However, a stronger correlation with mean 24-h systolic and diastolic blood pressure was seen in obstructive than central AHI (Figs. 2a, b).

**Table 1:** Baseline characteristics

	No SDB (*n* = 7)	CSA (*n* = 9)	OSA (*n* = 8)	*p* value
Age, years	49 ± 10	57 ± 8	54 ± 8	0.234
Body mass index (kg/m^2^)	28 ± 3	27 ± 3	28 ± 3	0.571
Male gender, *n* (%)	7 (100)	7 (78)	7 (88)	0.411
Heart rate (beats/minute)	81 ± 22	77 ± 16	85 ± 14	0.683
Systolic blood pressure (mmHg)	131 ± 28	122 ± 18	132 ± 20	0.551
Diastolic blood pressure (mmHg)	86 ± 12	75 ± 9	80 ± 12	0.188
Median 24 h systolic blood pressure (mmHg)	106 ± 10	111 ± 17	122 ± 17	0.177
Median 24 h diastolic blood pressure (mmHg)	68 ± 10	70 ± 8	76 ± 9	0.322
NTproBNP (pg/ml)	1212 (745)	1939 (2281)	854 (2531)	0.497
Hypertension, *n* (%)	5 (71)	4 (44)	6 (75)	0.364
Current smoker, *n* (%)	5 (71)	5 (56)	3 (38)	0.760
Diabetes mellitus, *n* (%)	1 (14)	2 (22)	2 (25)	0.871
Hypercholesterolaemia, *n* (%)	0 (0)	3 (33)	1 (13)	0.192
Pain-to-balloon-time (min)	291 (1155)	283 (231)	1068 (998)	0.148
NSTEMI (%)	1 (14)	0 (0)	1 (12)	0.553
TIMI flow grade 0 pre PCI, *n* (%)	4 (57)	7 (78)	7 (88)	0.388
TIMI flow grade 3 post PCI, *n* (%)	6 (86)	9 (100)	8 (100)	0.282
Thrombus aspiration, *n* (%)	4 (57)	3 (33)	3 (38)	0.605
Glycoprotein IIb/IIIa Inhibitor, *n* (%)	3 (43)	7 (78)	5 (63)	0.359
CK pre-PCI (U/l)	174 (1491)	342 (286)	487 (1559)	0.578
CK max (U/l)	2074 (2380)	1222 (3746)	2308 (3637)	0.981
Infarct size (%)	24 ± 12	26 ± 14	23 ± 15	0.896

Data are expressed as *n* (%), or mean ± standard deviation, or median [interquartile range]

*SDB* sleep-disordered breathing, *CSA* central sleep apnoea, *OSA*, obstructive sleep apnoea, *ACE* angiotensin-converting enzyme, *ADP* adenosine diphosphate, *CK* creatinine kinase, *PCI* percutaneous coronary intervention, *NT-proBNP* N-terminal probrain natriuretic peptide, *TIMI* thrombolysis in myocardial infarction

### Sleep characteristics

By definition, AHI was significantly lower in the no SDB group compared to the CSA and OSA groups (no SDB: 4 ± 2; CSA: 32 ± 22; OSA: 21 ± 7/h, *p* = 0.004) and the ratio of cAHI/AHI was > 50% in the CSA group and < 50% in the OSA group. The three groups were similar with respect to mean oxygen saturation, minimal oxygen saturation and time below SpO2 < 90% (Table 2).

**Table 2:** Nocturnal respiration and hypoxaemia

	No SDB (*n* = 7)	CSA (*n* = 9)	OSA (*n* = 8)	*p* value
Apnoea-hypopnoea index (episodes/h)	4 ± 2	32 ± 22	21 ± 7	**0.004**
cAHI/AHI (%)	51 ± 18	71 ± 12	22 ± 14	< 0.001*
AI/AHI (%)	32 ± 20	80 ± 12	42 ± 20	< 0.001*
Mean oxygen saturation (%)	92 ± 3	93 ± 2	94 ± 2	0.390
Minimal oxygen saturation (%)	86 ± 3	87 ± 4	85 ± 6	0.600

Data are expressed as mean ± standard deviation; or median [interquartile range]

*SDB* sleep-disordered breathing, *CSA* central sleep apnoea, *OSA* obstructive sleep apnoea, *cAHI* central apnoea-hypopnoea index, *oAHI* obstructive apnoea-hypopnoea index, *SpO2* oxygen saturation

*OSA vs. CSA *p* < 0.001, significant *p* values (*p* < 0.05) marked in bold

### Sphericity index

**Fig. 3:**
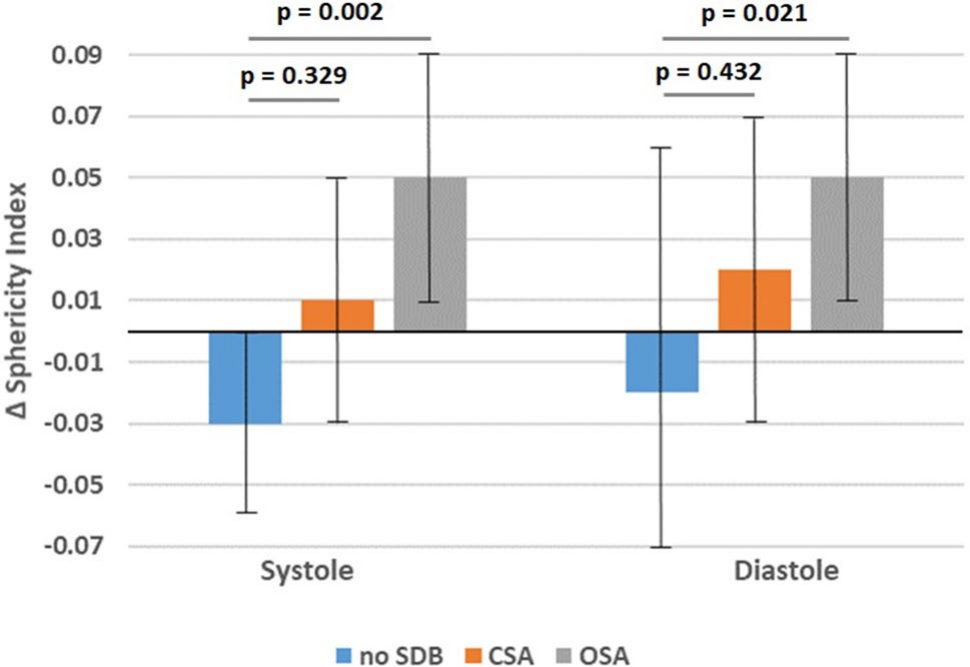
Bar chart of change in sphericity index from baseline to 12-weeks follow-up in systole and diastole. *SDB* sleep-disordered breathing, *CSA* central sleep apnoea, *OSA* obstructive sleep apnoea

At baseline, sphericity index was similar across the three groups. Conversely, at 12-weeks, systolic and diastolic sphericity index in the OSA group was significantly higher than in the no SDB group (0.21 ± 0.05 vs. 0.31 ± 0.07, *p* < 0.05; 0.31 ± 0.04 vs. 0.38 ± 0.05, *p* < 0.05; Table 3). Within 12 weeks after acute myocardial infarction, systolic sphericity index increased significantly in the OSA group compared to those without SDB, while patients with CSA exhibited a non-significant increase in systolic sphericity index (no SDB: − 0.03 ± 0.03; CSA: 0.01 ± 0.04; OSA: 0.05 ± 0.04, *p* = 0.002; Fig. 3). Similar results were observed in diastole (Fig. 3).

**Table 3:** Sphericity index

	No SDB (*n* = 7)	CSA (*n* = 9)	OSA (*n* = 8)	*p* value (ANOVA)
SI systolic baseline	0.24 ± 0.05	0.30 ± 0.08	0.26 ± 0.08	0.203
SI systolic follow-up	0.21 ± 0.05	0.31 ± 0.07	0.31 ± 0.07*	**0.008**
Delta SI systolic	− 0.03 ± 0.03	0.01 ± 0.04	0.05 ± 0.04*	**0.002**
*p* value _baseline—follow-up_	0.090	0.673	**0.004**	
SI diastolic baseline	0.32 ± 0.06	0.38 ± 0.07	0.33 ± 0.06	0.137
SI diastolic follow-up	0.31 ± 0.04	0.40 ± 0.07	0.38 ± 0.05*	**0.013**
Delta SI diastolic	− 0.02 ± 0.05	0.02 ± 0.05	0.05 ± 0.04*	**0.021**
*p* value _baseline—follow-up_	0.325	0.366	**0.005**	

Data are expressed as mean ± standard deviation

*SDB* sleep-disordered breathing, *CSA* central sleep apnoea, *OSA* obstructive sleep apnoea, *SI* sphericity index

*pairwise comparison OSA vs. no SDB *p* < 0.05, significant *p* values (*p* < 0.05) marked in bold

**Fig. 4:**
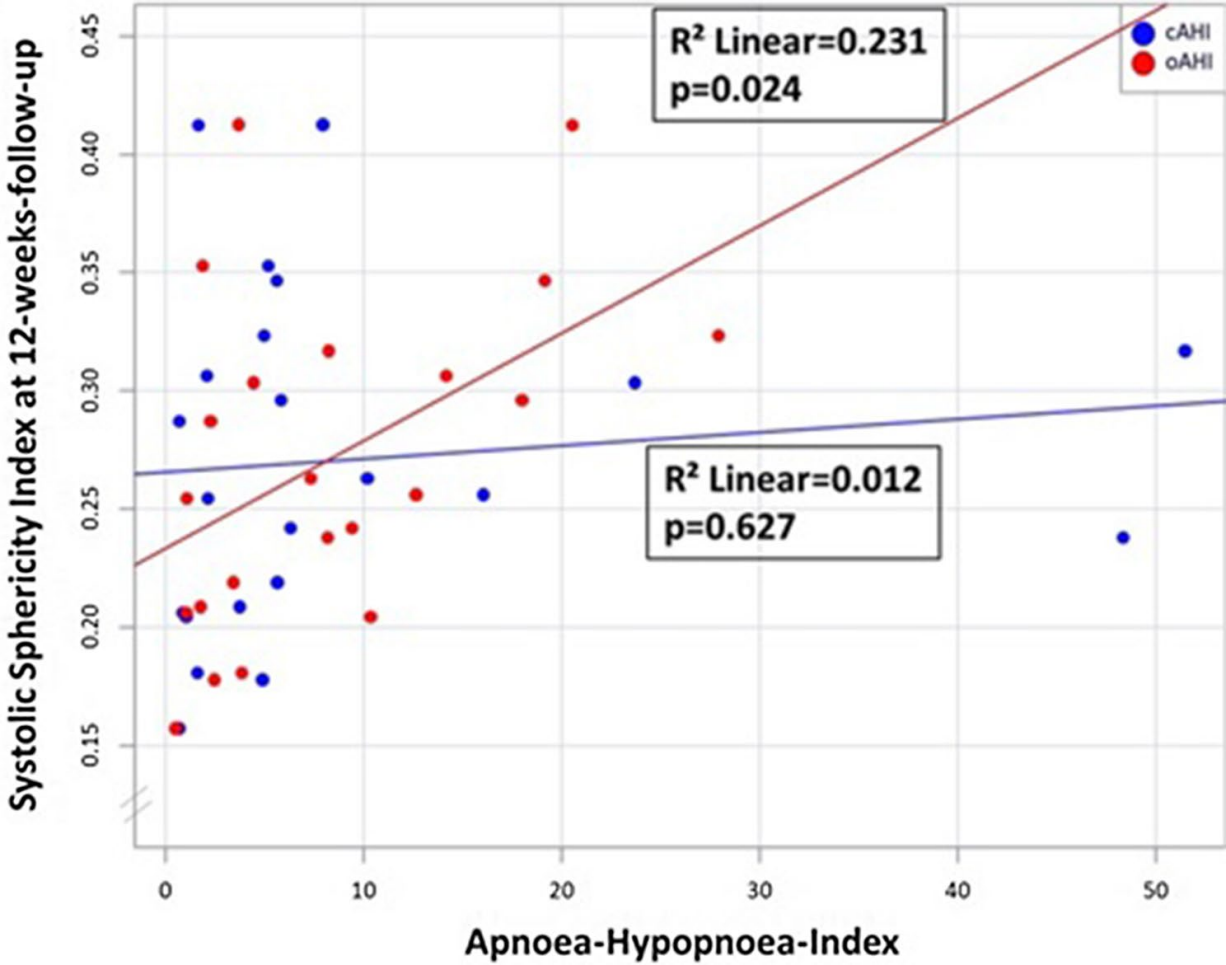
Scatter plot—sphericity index (systolic) 12 weeks after myocardial infarction and obstructive/central apnoea-hypopnoea index

In an unadjusted linear regression model, an increase in obstructive AHI was significantly associated with an increase in systolic sphericity index after 12-weeks follow-up, whereas an increase in central AHI was not (*R*^2^ = 0.231, *p* = 0.024; *R*^2^ = 0.012, *p* = 0.627; Fig. 4). These findings were robust in a multiple regression model accounting for age, sex and BMI (*p* = 0.046; *p* = 0.492), or adjusting for TIMI flow pre-percutaneous coronary intervention, infarct size, pain-to-ballon-time and systolic blood pressure (*p* = 0.040; *p* = 0.385; Table 4). Considering only apneas, the results were similar (Table 4).

**Table 4:** Regression analysis—obstructive/central respiratory events and systolic sphericity index [× 10^2^] 12 weeks after myocardial infarction

	Univariate analysis		Multiple analysis			
			Adjusted for age, sex and BMI		Adjusted for TIMI flow pre-PCI, infarct size, pain-to-ballon-time, systolic blood pressure	
	B (95% CI)	*p* value	B (95% CI)	*p* value	B (95% CI)	*p* value
oAHI	0.481 (0.068; 0.841)	**0.024**	0.459 (0.010; 0.858)	**0.046**	0.443 (0.021; 0.816)	**0.040**
cAHI	0.110 (− 0.179; 0.290)	0.627	0.187 (− 0.190; 0.379)	0.492	0.193 (− 0.134; 0.300)	0.385
oAI	1.460 (− 0.020; 2.940)	0.053	1.509 (− 0.258; 3.276)	0.089	1.991 (0.641; 3.340)	**0.007**
cAI	0.061 (− 0.174; 0.296)	0.594	0.102 (− 0.183; 0.388)	0.460	0.101 (− 0.129; 0.332)	0.366

*oAHI* obstructive apnoea-hypopnoea-index, *cAHI* central apnoea-hypopnoea-index, *oAI* obstructive apnoea-index, *cAI* central apnoea-index, *PCI* percutaneous coronary intervention, *TIMI* thrombolysis in myocardial infarction

Significant *p* values (*p* < 0.05) marked in bold

### Cardiac volumes

At baseline and follow-up, cardiac volumes were similar between the three groups (Table S2). Changes in left ventricular end-systolic and end-diastolic index from baseline to 12-weeks follow-up were significantly higher in the OSA group compared to the no SDB group (− 17 ± 15% vs. 11 ± 11%, *p* = 0.002; − 1 ± 13% vs. 15 ± 18%, *p* = 0.016, respectively). In contrast, the CSA group showed no statistically significant differences compared to the no SDB group (− 6 ± 15% vs. − 17 ± 15%, *p* = 0.395; 2 ± 15% vs. − 1 ± 13%, *p* = 0.467; Table 5). No significant differences were observed between groups for left ventricular mass index, stroke volume index or cardiac index (Table 5).

**Table 5:** Percentage change in cardiac volumes

	No SDB (*n* = 7)	CSA (*n* = 9)	OSA (*n* = 8)	*p* value (ANOVA)
LV mass index	− 17 ± 14	− 19 ± 15	− 14 ± 12	0.700
LV end-systolic volume index	− 17 ± 15	− 6 ± 15	11 ± 11*	**0.002**
LV end-diastolic volume index	− 1 ± 13	2 ± 15	15 ± 18*	**0.019**
Stroke volume index	− 1 ± 18	13 ± 20	24 ± 31	0.163
Cardiac index^a^	− 9 ± 13	4 ± 26	11 ± 32	0.344

Data are expressed as mean percentage change from baseline ± standard deviation

*SDB* sleep-disordered breathing, *CSA* central sleep apnoea, *OSA* obstructive sleep apnoea, *LV* left ventricular

Indexed to body surface area ^a^*n* = 1 in OSA missing, significant *p* values (*p* < 0.05) marked in bold, *pairwise comparison OSA vs. no SDB *p* < 0.05

### Wall thickness and left ventricular aneurysm

**Fig. 5:**
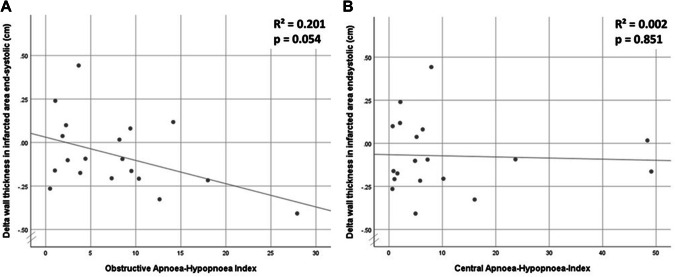
**a** Scatter plot—change in wall thickness in systole in infarcted area 12 weeks after myocardial infarction and obstructive apnoea-hypopnoea index. **b** Scatter plot—change in wall thickness in systole in infarcted area 12 weeks after myocardial infarction and central apnoea-hypopnoea index

Reduction in wall thickness was similar between groups in end-systole (no SDB: − 0.06 ± 0.19; CSA: − 0.04 ± 0.25; OSA: − 0.12 ± 0.20 *p* = 0.794) and end-diastole (no SDB: − 0.18 ± 0.18; CSA: − 0.08 ± 0.13; OSA: − 0.28 ± 0.37; *p* = 0.362). Correlation between obstructive AHI and change in wall thickness in the infarcted area was not significant (Fig. 5a; *R*^2^ = 0.201 *p* = 0.054). Central AHI was not correlated with a change in wall thickness (Fig. 5b; *R*^2^ = 0.002 *p* = 0.851). The frequency of left ventricular aneurysm was similar across groups [no SDB: 3 (43%); CSA: 6 (67%); OSA: 4 (50%); *p* = 0.645].

## Discussion

This study provides unique insights into the association between SDB and spheric cardiac remodelling after acute myocardial infarction: firstly, in contrast to the no SDB and CSA groups, patients with OSA exhibited a significant increase in systolic and diastolic sphericity index within 12 weeks after acute myocardial infarction. Secondly, the number of obstructive apnoeas and hypopnoeas was positively correlated with systolic sphericity index, whereas the number of central apnoeas and hypopnoeas was not. Data were robust after multiple regression analysis accounting for demographics and risk factors for spheric remodelling after acute myocardial infarction. Thirdly, no significant association between wall thickness, formation of cardiac aneurysm and SDB was observed.

To our best knowledge, an association between SDB in general and post-infarction spheric cardiac remodelling, measured by sphericity index, has not previously been described. After STEMI, up to 66% of patients are affected by OSA [23] and 12% by CSA [24]. Previous analyses in patients with acute myocardial infarction have revealed that the increase in systolic and diastolic left ventricular volumes is more pronounced in patients with SDB compared to those without. In patients with SDB no differences between OSA and CSA were observed [15]. Left ventricular function is diminished in both CSA and OSA patients after STEMI compared to patients without SDB [25–27] and an improvement in AHI over time is associated with an improvement in left ventricular function [24]. In OSA patients without acute myocardial infarction, higher AHI values are associated with increased cardiac wall thickness [28]. Data from patients with CSA are lacking. Thus, previous research has identified negative post-STEMI remodelling of the left ventricle in SDB. However, spheric remodelling, which has additional prognostic value [8], has not been specifically addressed.

### Effect size and potential clinical impact

The sphericity index can be used to detect cardiac remodelling with 100% sensitivity and 90% specificity and is more accurate than left ventricular volumes and ejection fraction [22]. A threshold of 0.29 in diastolic sphericity index is associated with a higher probability of developing heart failure [7, 29]. In the current analysis, this pathologic threshold was considerably exceeded at baseline, indicating clinically relevant spheric cardiac remodelling after myocardial infarction. Mannaerts et al. used 3D echocardiography to detect a sphericity index of 0.32 in patients with cardiac remodelling after STEMI compared to 0.22 in patients without cardiac remodelling, which is in line with our findings using CMR [22].

A more spherical left ventricle, assessed by ventriculography, is associated with reduced capacity and diminished ejection fraction. In addition, sphericity index is an independent predictor of survival in patients with coronary artery disease [30]. Survival after myocardial infarction is lower in patients with a higher sphericity index, which is used as a surrogate marker for congestive heart failure [8]. In this context, it is notable that the sphericity index increased considerably in our OSA cohort, which reflects potential detrimental effects on cardiac remodelling in this specific group of patients.

### Pathophysiology

The main pathophysiological differences between the CSA and OSA patients are negative intrathoracic pressure swings in the OSA group due to breathing efforts against the occluded pharynx, elevated blood pressure in the OSA cohort and arousals [31].

Data indicate that OSA-associated negative intrathoracic pressure swings contribute to increased left ventricular transmural pressure. In OSA patients with congestive heart failure, continuous positive airway pressure increases intrathoracic pressure [14], reduces left ventricular volume and left ventricular transmural pressure, impedes cardiovascular complications and improves cardiac function [11, 32–34]. Similar data in patients with CSA are lacking.

Moreover, the analysis evaluating the association between obstructive apnoeas and sphericity index showed higher numeric beta coefficients (effect size) compared to the analysis with obstructive apneas and hypopneas. This finding may underline, that in particular obstructive apnoeas and to a lower extend hypopnoeas may contribute to the intrathoracic negative pressure changes and might affect cardiac remodeling.

In addition, arousals from sleep may contribute to increased afterload and thus spheric cardiac remodeling. The association between the frequency of arousals may differ between obstructive and central respiratory events, because arousals occur in OSA to terminate apnoeas and activate pharyngeal muscles in order to re-open the occluded upper airways, while in CSA the association between arousals and respiratory events is modest [35]. In the present study we found a similar association between central and obstructive respiratory events with arousals from sleep (Figure S1a, b).

Activation of the sympathetic nervous system seems to be lower in CSA patients with congestive heart failure compared to those with OSA [36]. Several studies have revealed elevated blood pressure in OSA patients [37, 38]. This results in higher cardiac afterload and may lead to increased cardiac remodelling and a consequent deterioration in left ventricular mechanics [39]. In contrast, CSA has a relatively weak influence on blood pressure [12]. Our study also observed that higher blood pressures are more strongly associated with the severity of OSA, rather than CSA.

Thus, the increased left ventricular transmural pressure (negative intrathoracic pressure swings plus increased arterial blood pressure) in OSA patients, but not in CSA patients, may promote spheric cardiac remodelling and thinning of the left ventricular wall in the region of the myocardial infarction. Accordingly, in the present analysis, sphericity index is correlated with the quantity of obstructive apnoeas and hypopnoeas, but not central apnoeas and hypopnoeas. These results are robust, even after accounting for potentially confounding clinical factors such as age, sex and BMI.

The prevalence of sleep apnoea after myocardial infarction is high (approximately 54%) [40]. Within 12 weeks after myocardial infarction, the recovery of cardiac function is associated with a reduction in sleep apnoea, whereas severity of sleep apnoea is not changed in patients with persistent limited cardiac function [40]. Therefore, sleep apnoea should be re-evaluated when cardiac function changes.

### Limitations

The results from this sub-analysis must be interpreted in the light of several limitations. The sample size is relatively small, since only patients with infarcted left anterior descending artery were included. Furthermore, cardiac aneurysm is a rare event. However, the selected sample is appropriate considering that sphericity index was first evaluated in patients with anterior wall myocardial infarction. A direct causal relationship cannot be inferred due to the observational nature of the study design. Neither intrathoracic pressures nor oesophageal pressures were recorded to quantify the postulated negative intrathorathic pressure swings during obstructive apneas [31]. Larger, prospective, randomised trials are now required to verify these findings. Further data will be generated by the interventional TEAM-ASV I study (NCT02093377, adaptive servoventilation versus control in patients with acute myocardial infarction and sleep-disordered breathing).

## Conclusion

In contrast to CSA and no SDB, OSA is associated with spheric cardiac remodelling within the first 12 weeks after acute myocardial infarction. Data suggest that OSA-related negative intrathoracic pressure swings may contribute to spheric cardiac remodelling after acute myocardial infarction. Findings add an argument that differentiation of OSA and CSA is needed in patients with acute myocardial infarction.

## Electronic supplementary material

Below is the link to the electronic supplementary material.

Supplementary file 1 (DOCX 122 kb)
