# Supplementary material for: Obstructive sleep apnoea but not central sleep apnoea is associated with left ventricular remodelling after acute myocardial infarction
Source: Clin Res Cardiol. 2020 Jun 9;110(7):971–82. doi: 10.1007/s00392-020-01684-z (PMC8238704; doi:10.1007/s00392-020-01684-z)
Supplement: Supplementary file 1 — (DOCX 122 kb) [file 392_2020_1684_MOESM1_ESM.docx]

**Supplementary material**

This appendix is provided by the authors to give additional information about the research.

Supplement to: **Obstructive sleep apnoea but not central sleep apnoea is associated with left ventricular remodelling after acute myocardial infarction**

Christoph Fisser^1^; Kristina Götz^1^; Andrea Hetzenecker^2^; Kurt Debl^1^; Florian Zeman^3^; Okka W Hamer^4^; Florian Poschenrieder^4^; Claudia Fellner^4^; Stefan Stadler^1^; Lars S Maier^1^; Michael Pfeifer^2^; Stefan Buchner^1,5^; Michael Arzt^1^

^1^ Department of Internal Medicine II, University Medical Centre Regensburg, Regensburg, Germany

^2^ Department of Pneumology, Donaustauf Hospital, Donaustauf, Germany

^3^ Centre for Clinical Studies, University Medical Centre Regensburg, Regensburg, Germany

^4^ Department of Radiology, University Medical Centre Regensburg, Regensburg, Germany

^5^ Department of Internal Medicine, Cham Hospital, Cham, Germany

Corresponding Author:

Christoph Fisser, MD

Department of Internal Medicine II, University Medical Centre Regensburg

Franz-Josef-Strauß-Allee 11, 93053 Regensburg, Germany

Phone: ++49 941 944 7281

Facsimile: ++49 941 944 7282

Email: [Christoph.Fisser@ukr.de](mailto:Christoph.Fisser@ukr.de)

**Table S1: Comparison of mean 24-hour systolic and diastolic blood pressure**

| Mean 24-hour blood pressure (mmHg) | CSA  (n=9) | OSA  (n=6) | p value |
| --- | --- | --- | --- |
| Systolic | 111±17 | 122±17 | 0.229 |
| Diastolic | 70±8 | 76±6 | 0.205 |

Data are expressed as mean ± standard deviation, CSA, central sleep apnoea; OSA obstructive sleep apnoea.

**Table S2: Cardiac volumes indexed to body surface area at baseline and 12-weeks follow-up**

|  | No SDB  (n=7) | CSA  (n=9) | OSA  (n=8) | p value | p_CSA vs. no SDB_ | p_OSA vs. no SDB_ | p_CSA vs. OSA_ |
| --- | --- | --- | --- | --- | --- | --- | --- |
| **Baseline** |  |  |  |  |  |  |  |
|  |  |  |  |  |  |  |  |
| LV mass index | 78±9 | 80±21 | 80±16 | 0.963 | 1.000 | 1.000 | 1.000 |
| LVESV index | 43±10 | 50±20 | 39±8 | 0.269 | 0.964 | 1.000 | 0.345 |
| LVEDV index | 85±14 | 85±25 | 72±10 | 0.267 | 1.000 | 0.5.13 | 0.460 |
| Stroke volume index | 42±8 | 35±8 | 33±7 | 0.067 | 0.201 | 0.083 | 1.000 |
| Cardiac index^a^ | 2.7±0.4 | 2.5±0.6 | 2.3±0.40 | 0.315 | 1.000 | 0.406 | 1.000 |
| **Follow-up** |  |  |  |  |  |  |  |
| LV index | 64±11 | 64±15 | 68±11 | 0.771 | 1.000 | 1.000 | 1.000 |
| LVESV index | 35±9 | 46±17 | 43±7 | 0.243 | 0.300 | 0.780 | 1.000 |
| LVEDV index | 76±11 | 84±19 | 82±8 | 0.501 | 0.746 | 1.000 | 1.000 |
| Stroke volume index | 41±6 | 39±7 | 39±7 | 0.737 | 1.000 | 1.000 | 1.000 |
| Cardiac index^a^ | 2.5±0.4 | 2.5±0.4 | 2.5±0.7 | 0.980 | 1.000 | 1.000 | 1.000 |
|  |  |  |  |  |  |  |  |

Data are expressed as mean ± standard deviation, SDB, sleep-disordered breathing; CSA, central sleep apnoea; OSA obstructive sleep apnoea; CMR: cardiac magnetic resonance imaging; LV: left ventricular; LVESV, left ventricular end-systolic volume; LVEDV, left ventricular end-diastolic volume; ^a^ n=1 in OSA missing; significant p values (p < 0.05) marked in bold; index: indexed to body surface area.

**Figure S1a: Scatter plot – Aoursal index per hour at baseline and obstructive apnoea-hypopnoea index**


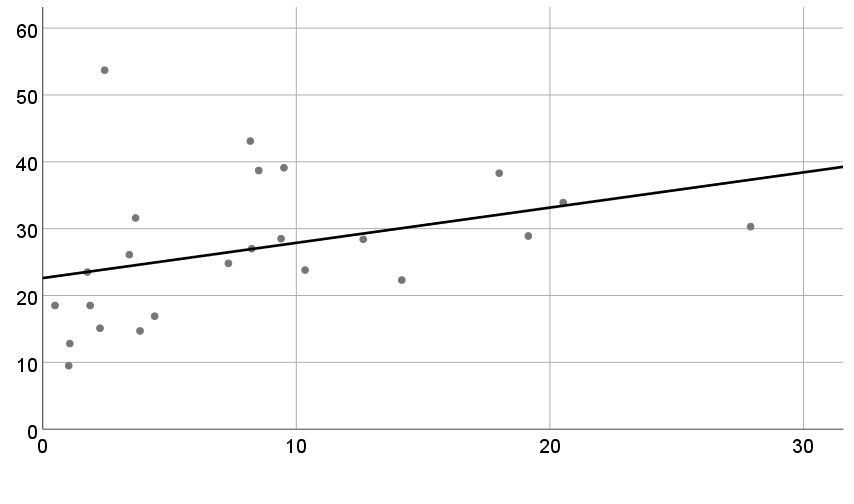


Obstructive Apnoea-Hypopnoea-Index /h

Arousal index /h at baseline

**Figure S1a: Scatter plot – Aoursal index per hour at baseline and central apnoea-hypopnoea index**


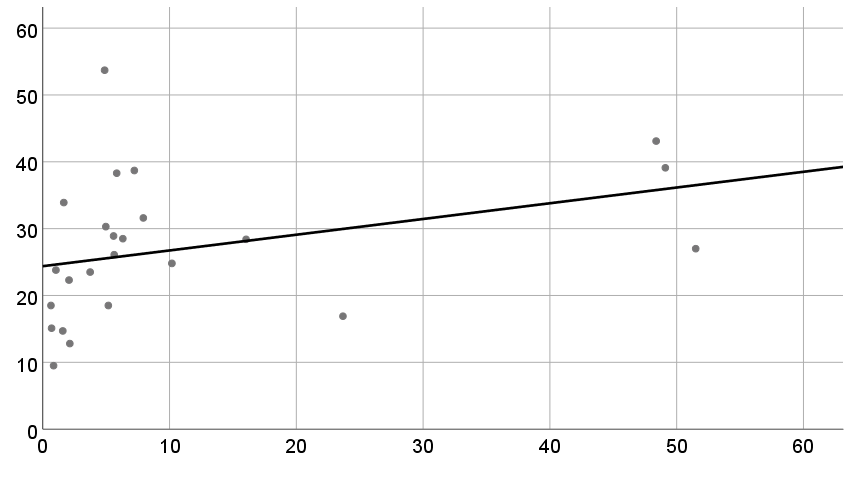


Arousal index /h at baseline

Central Apnoea-Hypopnoea-Index /h
